# Adalimumab-Associated Sensory Neuropathy in a Young Patient With Spondyloarthritis: A Complication of Anti-tumor Necrosis Factor Therapy

**DOI:** 10.7759/cureus.100885

**Published:** 2026-01-05

**Authors:** Sushma Konnepati, Vaibhav Ingle, Akash Pawar, Firoz Khan, Agrata Sharma

**Affiliations:** 1 Department of General Medicine, All India Institute of Medical Sciences, Madurai, IND; 2 Department of General Medicine, All India Institute of Medical Sciences, Bhopal, IND; 3 Department of Medicine, All India Institute of Medical Sciences, Bhopal, IND; 4 Department of Neurology, All India Institute of Medical Sciences, Bhopal, IND

**Keywords:** adalimumab, anti-tnf agents, drug-induced neuropathy, painful sensory motor peripheral neuropathy, spondyloarthritis

## Abstract

Demyelinating neurological diseases are important contraindications to initiating anti-tumor necrosis factor (TNF) biologic therapy; however, these agents can also rarely induce demyelinating conditions, including neuropathies. This immunological breakdown induced by anti-TNF drugs is a class effect and may be reversible upon discontinuation of the drug. In this report, the authors have presented an anti-TNF-induced neuropathy in an ankylosing spondylitis patient that reversed on drug discontinuation. With more frequent clinical use of anti-TNF drugs, especially with the advent of biosimilars, their association with demyelinating diseases may become more discernible. Reintroduction of the above medicines in the given scenario is not advisable. Host factors predisposing to the above adverse effect need more research.

## Introduction

Biological agents, such as tumor necrosis factor-alpha (TNF-α), are recommended for inflammatory arthritis, including rheumatoid arthritis and spondyloarthritis, particularly in patients who are unresponsive to conventional synthetic disease-modifying antirheumatic drugs (csDMARDs) such as methotrexate, sulfasalazine, leflunomide, and hydroxychloroquine. These agents not only improve functional outcomes but also halt radiological progression. Although they have a good safety profile, anti-TNF agents are associated with various adverse events. These range from opportunistic infections (e.g., tuberculosis, herpes zoster) and autoimmune conditions (e.g., drug-induced lupus erythematosus) to malignancies and, less commonly, neurological complications, such as central and peripheral demyelinating disorders. However, their increasing use during the last decade has revealed a variety of immune-mediated adverse events [1]. The clinical spectrum of neuropathy is heterogeneous, as evidenced by many case reports and case series, presenting as axonal, symmetric, sensory-motor polyradiculoneuropathy or as mononeuropathy multiplex or simplex, with conduction block. Demyelination associated with anti-TNF-α agents appears to be reported more commonly as involving the central nervous system (CNS) rather than the peripheral nervous system (PNS) [2]. Although rare, peripheral neuropathy is an emerging and potentially debilitating adverse event that warrants clinical vigilance. It poses a diagnostic challenge whether neuropathy is drug-induced or a part of a disease, such as a vasculitic phenomenon. The response to treatment also varies; some improve with withdrawal of the offending agent, whereas some may require high-dose steroids, plasmapheresis, or intravenous immunoglobulin (IVIG). So, a high index of clinical suspicion is needed. We present a case of adalimumab-induced sensory neuropathy in a young male with spondyloarthritis.

## Case presentation

An 18-year-old male presented to our rheumatology clinic with inflammatory back pain and peripheral arthritis for the last three years. On evaluation, X-ray of the lumbosacral spine showed grade 3 bilateral sacroiliitis. He also tested positive for HLA B27. Diagnosis of axial spondyloarthritis was made based on the presence of chronic inflammatory back pain with radiographic evidence of sacroiliitis and genetic predisposition in the form of HLA B27 positivity (Assessment of SpondyloArthritis International Society {ASAS} classification criteria). Initial management included non-steroidal anti-inflammatory agents at inflammatory doses (etoricoxib 90 mg once daily for three weeks, followed by indomethacin sustained release (SR) 75 mg twice daily for the next two weeks due to suboptimal response with the former) and sulfasalazine 500 mg twice daily. Due to inadequate clinical response and persistently high disease activity, biologicals were considered, and adalimumab 40 mg injection was administered subcutaneously every two weeks. He clinically improved, and disease activity scores improved. However, following the sixth dose, the patient developed bilateral tingling and burning sensations in the lower limbs. He, however, had no clinical symptoms of motor involvement. Neurological examination revealed minimal weakness in the proximal and distal lower limbs (4+/5 on the Medical Research Council {MRC} grading). Sensory examination revealed impaired pin prick up to below the knees in the right limb and up to the mid shin in the left lower limb. Romberg's test was negative, and vibration/proprioception was intact. Deep tendon reflexes were hyporeflexic, and bilateral plantar responses were flexor. Neurological examination of the bilateral upper limbs and cranial nerves was normal.

Investigations

Routine blood investigations, including serum glucose, serum vitamin B12, and thyroid function, were all within normal limits (except for C-reactive protein/erythrocyte sedimentation rate, and rheumatoid factor), and infectious disease screening for HIV, hepatitis B and C, and latent tuberculosis infection were negative (Table 1). Motor nerve conduction studies (NCS) demonstrated a prolonged distal latency in bilateral ulnar and tibial nerves and decreased conduction velocity in the right tibial nerve, and sensory NCS demonstrated reduced conduction velocity in bilateral median and right ulnar nerves, indicative of a sensorimotor polyneuropathy (Table 2). Lumbar puncture for CSF analysis was refused by the patient. Given the early clinical stage and limited diagnostic yield, a nerve biopsy was not performed. The close temporal association with adalimumab administration and the absence of alternative etiologies strongly suggested a drug-induced neuropathy.

**Table 1:** Relevant investigations of the patient with reference values. TSH: thyroid-stimulating hormone; RF: rheumatoid arthritis; CCP: cyclic citrullinated peptide; ANA: anti-nuclear antibody; ANCA: anti-neutrophil cytoplasmic antibodies; IFA: immunofluorescence assay; HBsAg: hepatitis B surface antigen; HCV: hepatitis C virus; TB: tuberculosis

Investigations	Value	Reference range
Hemoglobin (g/dL)	12	11-16
Platelet count (per µL)	342000	150000-450000
Urea (mg/dL)	22	13-40
Serum creatinine (mg/dL)	0.7	0.6-1.2
HbA1c (%)	5.4	<6.5
Serum TSH (µU/mL)	1.854	0.5-5
Serum vitamin B12 (pg/mL)	262	200-900
CRP (mg/L)	78	<5
ESR (mm/h)	54	0-20
RF (U/mL)	9.62	<9.6
Anti-CCP (U/mL)	Negative	<5
ANA (IFA)	Negative	Negative
ANCA (IFA)	Negative	Negative
Anti-HIV	Negative	Negative
HBsAg	Negative	Negative
Anti-HCV	Negative	Negative
Latent TB testing (TB QuantiFERON)	Negative	Negative

**Table 2:** Nerve conduction studies of upper and lower limbs. NCV: nerve conduction velocity

Site/nerve	Latency (ms)	Duration (ms)	Amplitude (mV)	Distance (mm)	Nerve conduction velocity (m/s)	Reference range
Left median - elbow	3.1	6.2	11.2	240	53.3	Latency ≤4.0 ms; NCV ≥50 m/s
Right median - elbow	3.0	6.1	8.4	240	52.7	Latency ≤4.0 ms; NCV ≥50 m/s
Left ulnar - below elbow	10.2	5.3	8.5	240	49.5	Latency ≤3.5 ms; NCV ≥50 m/s
Left ulnar - above elbow	11.6	5.7	9.4	70	48.3	Latency ≤3.5 ms; NCV ≥50 m/s
Right ulnar - below elbow	11	6.8	7.4	240	51.1	Latency ≤3.5 ms; NCV ≥50 m/s
Right ulnar - above elbow	11.8	6.7	10.1	70	82.4	Latency ≤3.5 ms; NCV ≥50 m/s
Left peroneal - head of fibula	12.5	9.5	3.9	330	45.2	Latency ≤6.5 ms; NCV ≥40-50 m/s
Left peroneal - popliteal	14.3	9.5	3.9	70	38.9	Latency ≤6.5 ms; NCV ≥40-50 m/s
Right peroneal - head of fibula	13	8.7	4.2	330	40.0	Latency ≤6.5 ms; NCV ≥40-50 m/s
Right peroneal - popliteal	15.3	8.8	4.0	70	30.4	Latency ≤6.5 ms; NCV ≥40-50 m/s
Left tibial - popliteal	17.5	7.5	5	400	38.6	Latency ≤6.0 ms; NCV ≥40-50 m/s
Right tibial - popliteal	18.2	7.0	9.6	400	33.1	Latency ≤6.0 ms; NCV ≥40-50 m/s

Treatment

Adalimumab was discontinued immediately upon suspicion of drug-induced neurotoxicity. The patient was managed symptomatically with pregabalin 75 mg once daily and occasional short-acting opioids for neuropathic pain. No additional immunomodulatory therapy was initiated. Progressive symptomatic improvement was observed over the ensuing weeks.

Outcome and follow-up

Within four weeks of adalimumab discontinuation, the patient experienced partial resolution of neuropathic symptoms. He remained under close rheumatological and neurological follow-up. To mitigate the risk of relapse, the Janus kinase inhibitor tofacitinib was being considered as an alternative therapeutic option.

## Discussion

Tumor necrosis factor-alpha (TNF-α) is a pleiotropic cytokine essential to immune regulation and inflammatory responses. It is predominantly produced by macrophages and plays a key role in autoimmune diseases. TNF inhibitors, such as adalimumab, act by reducing pro-inflammatory cytokines (IL-1, IL-6), chemokines (IL-8), and acute-phase reactants, such as CRP, while also modulating vascular function, leucocyte trafficking, and T-cell phenotypes [3,4]. Infliximab, golimumab, adalimumab, and certolizumab are monoclonal antibodies specific to TNF, whereas etanercept is a fusion protein that binds to TNF and lymphotoxin alpha. Adalimumab, a fully human monoclonal IgG1 antibody that targets TNF-α, is administered subcutaneously at 40 mg every two weeks.

Despite their therapeutic efficacy, TNF inhibitors can rarely trigger paradoxical immune-mediated effects, including demyelinating disorders. The proposed mechanisms include immune dysregulation, molecular mimicry, or reactivation of latent conditions [3]. Reported neuropathies associated with TNF inhibitors include Guillain-Barré syndrome, chronic inflammatory demyelinating polyneuropathy (CIDP), multifocal motor neuropathy, and axonal variants [5,6].

All TNF inhibitors have been associated with such neurological events, and there is no evidence supporting one agent as safer than others. Neurological symptoms may develop months to years after treatment initiation. In the present case, symptoms appeared within three months, which is relatively early [7,8]. Although a definitive causal link is difficult to establish, the chronology of events, lack of alternative explanations, and symptom resolution after discontinuation support a likely drug-induced neuropathy. Rechallenge studies have shown recurrence, implying immunological priming [9]. Discontinuation of the drug typically results in partial or complete resolution of symptoms, sometimes requiring corticosteroids, intravenous immunoglobulin (IVIG), or plasmapheresis [8]. In our patient, symptoms resolved with drug withdrawal alone.

Peripheral neuropathies may also occur in rheumatic diseases due to other causes like vasculitis, entrapment, nutritional deficiencies, or drug toxicity. Nonetheless, the absence of disease activity and other identifiable causes in our case strongly suggests adalimumab as the trigger [6,7]. A study reported a median onset time of 16.8 months for TNF inhibitor-induced neuropathy, although earlier occurrences have been documented [8]. Whether it is feasible to rechallenge with anti-TNF remains controversial. Limited evidence suggests a possible dose-related effect, necessitating cautious risk-benefit assessment [10,11]. In a systematic review of 101 patients with anti-TNF-associated polyneuropathy, motor-predominant involvement with complete recovery (with anti-TNF discontinuation) was observed in approximately one-third of cases. Still, symptoms recurred in seven re-exposed patients [12]. Predominant neurophysiology was conduction block.

This case underscores the importance of maintaining a high index of suspicion for neurological complications in patients receiving anti-TNF therapy. Timely recognition and discontinuation can prevent progression and lead to full recovery.

## Conclusions

In conclusion, while anti-TNF-α agents remain a cornerstone in the management of several chronic inflammatory diseases, clinicians must remain vigilant about their rare but potentially serious neurological complications. Peripheral demyelinating neuropathies may develop shortly after treatment initiation, and their early recognition is crucial to prevent long-term disability. Prompt discontinuation of the offending drug can lead to significant recovery and, in many cases, may be sufficient. For patients with severe or progressive disease, escalation to immunomodulatory interventions such as corticosteroids, intravenous immunoglobulin, or plasma exchange can be helpful. Ultimately, balancing the benefits of anti-TNF-α therapy with awareness of these rare adverse events ensures better patient outcomes through individualized, cautious, and well-monitored use.
